# Long-term dystrophin restoration supports development of splice correction therapy for DMD patients with exon 2 duplications

**DOI:** 10.1016/j.omtm.2023.101160

**Published:** 2023-11-24

**Authors:** Thomas C. Roberts

**Affiliations:** 1 Department of Paediatrics, University of Oxford, Oxford OX3 7TY, UK; 2 Institute of Developmental and Regenerative Medicine, University of Oxford, IMS-Tetsuya Nakamura Building, Oxford OX3 7TY, UK; 3 MDUK Oxford Neuromuscular Centre, Oxford OX3 7TY, UK

## Main text

Duchenne muscular dystrophy (DMD) is a monogenic disorder caused by the absence of the dystrophin protein, which acts to protect skeletal and cardiac muscle from contractile damage. The field of advanced therapeutics for DMD has been particularly productive, with regulatory approvals achieved for four splice-correcting antisense oligonucleotides, one gene therapy (all approved by the US Food and Drug Administration [FDA]), and one stop codon readthrough compound (approved by the European Medicines Agency).^1^ Despite these high-profile successes, it is generally regarded that the clinical challenge for DMD is far from met, with these drugs exhibiting only limited therapeutic benefit or having applicability to only subsets of DMD patients.

**Figure 1:**
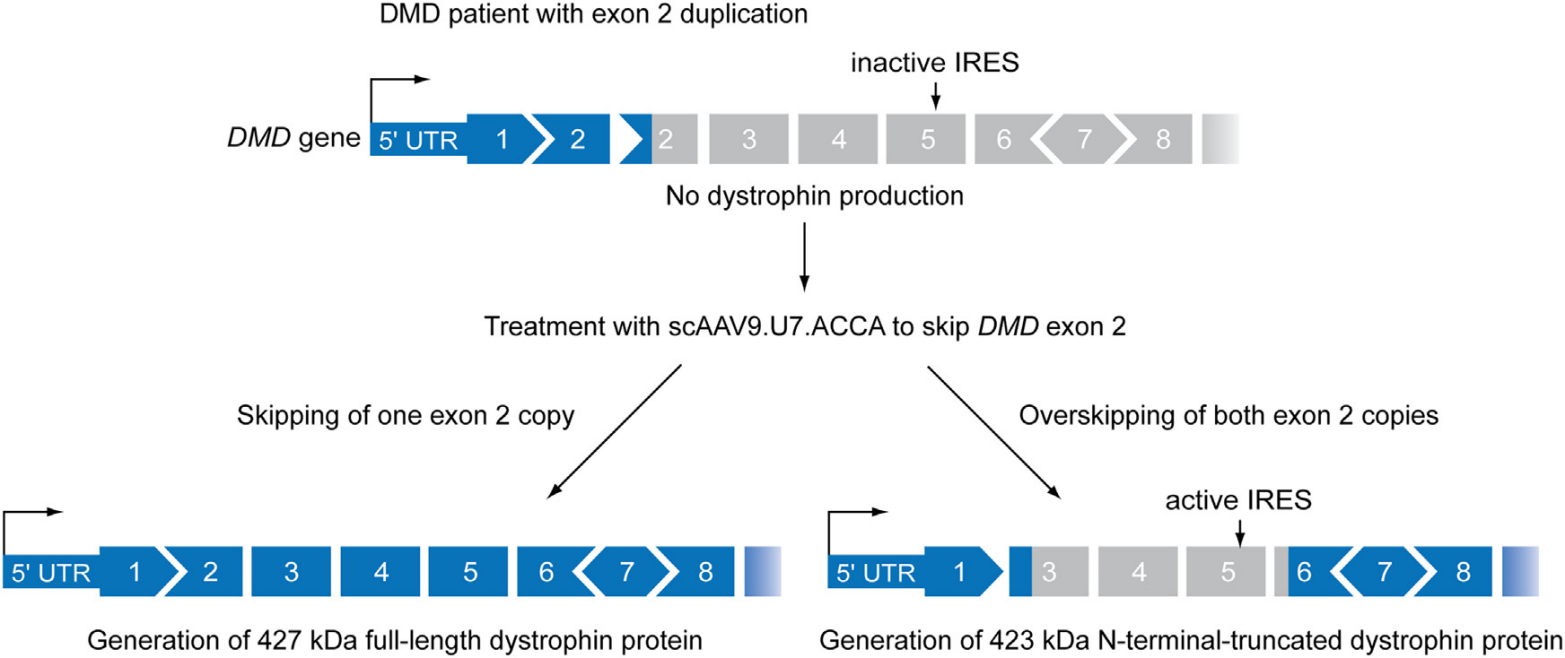
Splicing outcomes in the treatment of patients with duplications of *DMD* exon 2 A region of the *DMD* gene for a patient carrying a duplication of exon 2. In the context of exon 2 duplication, the IRES sequence in exon 5 is inactive. Thick blue boxes indicate in-frame coding exons. Gray boxes indicate out-of-frame exons. The shape of the exons depicts how the triplet base code is distributed across the exons such that the shapes must fit together to generate an in-frame coding transcript. Therapeutic skipping of exon 2 mediated by treatment with scAAV.U7.ACCA results in 2 possible outcomes. Skipping a single copy of exon 2 results in the generation of full-length 427-kDa dystrophin protein. Conversely, “overskipping,” whereby both copies of exon 2 are excised from the mature DMD transcript leads to premature termination in exon 3. However, an active IRES site in exon 5 drives cap-independent translation of an N-terminally truncated 423-kDa dystrophin protein with a start codon located in exon 6. As such, both splicing outcomes are therapeutically beneficial.

DMD is caused by a plethora of diverse mutations, with whole-exon deletions being the most common. Conversely, whole-exon duplications account for ∼11% of disease-causing variants, of which duplication of *DMD* exon 2 constitutes ∼10%.^2^ Genetic correction in such exon duplication patients presents both challenges and opportunities. Splice-switching therapies have been applied widely to the *DMD* gene because in many cases, exon-skipped dystrophin transcripts encode for partially functional pseudodystrophin proteins that are associated with milder disease pathology. Removal of the duplicated exon by splice switching would be desirable, although such technologies have no easy way of distinguishing between the original or duplicated exon, such that either one or both exons may be removed from the mature *DMD* transcript following splice correction. It is unique that both possible splicing outcomes are potentially beneficial in the case of *DMD* exon 2 duplications, because the removal of one copy of exon 2 will generate full-length healthy dystrophin mRNA, whereas the removal of both copies of exon 2 results in cap-independent translation initiation from an internal ribosome entry site (IRES) located in exon 5, with the corresponding alternate start codon located in exon 6. This alternative translation product is a highly functional, N-terminal-truncated dystrophin proteoform lacking a portion of the actin binding domain 1^3^ (Figure 1). Expression of this N-terminal-truncated dystrophin has been reported to result in a very mild form of Becker muscular dystrophy, whereby ambulation was maintained until at least the 7^th^ decade of life.^4^ Interestingly, the duplication of exon 2 is associated with the loss of function of the exon 5 IRES sequence.^3^

The restoration of full-length dystrophin is currently beyond the reach of most other gene/oligonucleotide therapies for DMD, because these rely either on the generation of partially functional internally deleted pseudodystrophins as a consequence of exon skipping, or by design in the case of microdystrophin minigene therapies (in which viral vector packaging capacity is limiting). Although it is presumed that such shortened dystrophins can function sufficiently to prevent dystrophic pathology, the restoration of full-length dystrophin is clearly preferable.

In the present issue of *Molecular Therapy - Methods & Clinical Development*, Gushchina et al. have provided new evidence that skipping exon 2 in Dup2 mice (which carry a *Dmd* exon 2 duplication)^5^ using an adeno-associated virus (AAV)–delivered expressed splice correction strategy results in the long-term rescue of dystrophin protein expression (i.e., up to 18 months posttreatment).^6^ The treatment (known as scAAV9.U7.ACCA) consists of a self-complementary AAV9 vector encoding four modified U7 small nuclear RNA (snRNA) antisense RNA expression cassettes^7^ designed to induce *Dmd* exon 2 skipping via the targeting of splice acceptor and splice donor sites.^3^

Dup2 mice (3 months old) were intravenously injected with 3 × 10^13^ vg/kg (a previously determined minimally efficacious dose)^8^ and animals harvested at 21 months of age.^6^ The generation of therapeutically skipped *Dmd* mRNAs (both single and double exon 2 skipped) was detected by RT-PCR, which determined that skipping was most efficient in the heart (∼73%), with levels of ∼46% in tibialis anterior (TA) and ∼32% in diaphragm. These findings were mirrored in protein level assessments of dystrophin restoration. Western blot analysis showed that dystrophin was expressed at ∼65% in heart, ∼42% in TA, and ∼18% in diaphragm (relative to wild-type controls). Treated Dup2 mice harvested at 18 months posttreatment demonstrated improved force output in TA and diaphragm, which did not reach the levels observed in wild-type controls. Similarly, eccentric contraction-induced force was partially rescued in treated TA muscles. Systemic administration of scAAV9.U7.ACCA also resulted in the normalization of mean myofiber diameter to wild-type levels in both TA and diaphragm, although levels of central nucleation were slightly increased following treatment. These data demonstrate that therapy with scAAV9.U7.ACCA to induce *Dmd* exon 2 skipping promotes the long-term expression of relatively high levels of dystrophin protein across therapeutically relevant tissues, together with partial functional correction and improvements in muscle histopathology.^6^

These findings build on extensive previous work from Kevin Flanigan’s group, demonstrating proof-of-concept,^3^^,^^9^ dose finding,^8^ off-target splicing analysis,^10^ and favorable toxicity profiles in nonhuman primates.^11^ Most notably, the scAAV9.U7.ACCA-mediated exon 2 skipping therapeutic strategy has progressed to the clinical trial stage (Phase I/II, NCT04240314). The DMD research community is eagerly awaiting the reporting of findings from this trial, although preliminary (non-peer-reviewed) results are highly promising.^12^ Specifically, three DMD boys have been treated with scAAV9.U7.ACCA vectors with levels of 70% of normal dystrophin reported, and 99% of myofibers being dystrophin positive, as assessed in 4-month posttreatment biopsy material.^12^ These findings are notable for two important reasons. First, this therapeutic intervention is the first gene therapy to generate full-length dystrophin in DMD patient muscle. Second, one of the trial participants is the youngest ever DMD boy to receive gene therapy, with preliminary evidence suggesting that dystrophin restoration effectiveness is inversely related to age at treatment.^12^

In contrast with the approved exon skipping antisense compounds that require repeat administration, the scAAV9.U7.ACCA approach is a one-time treatment that relies on the retention of episomal AAV genomes in the myonuclei of treated patient muscles. The accretion of new myonuclei during normal muscle growth and development may result in a dilution effect, whereby the proportion of dystrophin-producing myonuclei become progressively reduced over time. Similarly, AAV genomes may be lost from muscle if the containing regions undergo necrosis, or may be “functionally lost” if the viral DNA accumulates silent-state chromatin modifications and/or cytosine 5-methylation. As such, the demonstration of persistent dystrophin expression and functional improvement 18 months posttreatment is highly encouraging. Notably, the functional correction observed at the 18-month time point reported in the study by Gushchina et al. is similar to that reported previously reported for 3- or 6-month posttreatment time points,^9^ which is indicative of a sustained level of partial phenotypic rescue.^6^

Although highly promising, it is important to emphasize that the exon 2 skipping approaches would be applicable to only ∼1% of all DMD patients. However, if it can be demonstrated that there are major benefits to restoring full-length dystrophin versus internally deleted, exon skipped pseudodystrophins or microdystrophin transgenes, then there will likely be important implications for the field of DMD therapeutics more generally. Similarly, the optimal timing of treatment initiation in DMD patients is currently unknown (and in many cases is restricted by diagnostic delay), such that findings from the scAAV9.U7.ACCA clinical trial may be informative in relation to this important issue. Notably, the FDA approval of delandistrogene moxeparvovec (Elevidys), an AAV-encoded microdystrophin gene therapy, presents a potential competitive challenge to AAV-encoded exon skipping approaches because the former strategy is theoretically applicable to a much larger cohort of potential patients. However, the dose of AAV used in the study by Gushchina et al. is almost 10-fold lower than those typically used for microdystrophin therapy (∼2 × 10^14^ vg/kg), a significant advantage given the potential dangers of high-dose AAV therapies.^1^

The study by Gushchina et al. provides additional preclinical evidence of the long-term effectiveness of the scAAV9.U7.ACCA-mediated exon 2 skipping approach. Whether such durable effects will be similarly observed in human patients is unknown and can only be determined by long-term follow-up of clinical trial participants at this stage.
